# Accelerated oral nanomedicine discovery from miniaturized screening to clinical production exemplified by paediatric HIV nanotherapies

**DOI:** 10.1038/ncomms13184

**Published:** 2016-10-21

**Authors:** Marco Giardiello, Neill J. Liptrott, Tom O. McDonald, Darren Moss, Marco Siccardi, Phil Martin, Darren Smith, Rohan Gurjar, Steve P. Rannard, Andrew Owen

**Affiliations:** 1 Department of Chemistry, University of Liverpool, Crown Street, Liverpool L69 7ZD, UK; 2 Department of Molecular and Clinical Pharmacology, University of Liverpool, Block H, 70 Pembroke Place, Liverpool L69 3GF, UK

## Abstract

Considerable scope exists to vary the physical and chemical properties of nanoparticles, with subsequent impact on biological interactions; however, no accelerated process to access large nanoparticle material space is currently available, hampering the development of new nanomedicines. In particular, no clinically available nanotherapies exist for HIV populations and conventional paediatric HIV medicines are poorly available; one current paediatric formulation utilizes high ethanol concentrations to solubilize lopinavir, a poorly soluble antiretroviral. Here we apply accelerated nanomedicine discovery to generate a potential aqueous paediatric HIV nanotherapy, with clinical translation and regulatory approval for human evaluation. Our rapid small-scale screening approach yields large libraries of solid drug nanoparticles (160 individual components) targeting oral dose. Screening uses 1 mg of drug compound per library member and iterative pharmacological and chemical evaluation establishes potential candidates for progression through to clinical manufacture. The wide applicability of our strategy has implications for multiple therapy development programmes.

## Abstract (web summary)

Nanomedicine efficacy in a clinical setting depends on the pharmacological properties of the therapeutic nanoparticles. Here, the authors exemplify an accelerated translational strategy from small-scale screening to clinical scale-up for an orally-dosed aqueous paediatric HIV nanomedicine.

## Graphical abstract

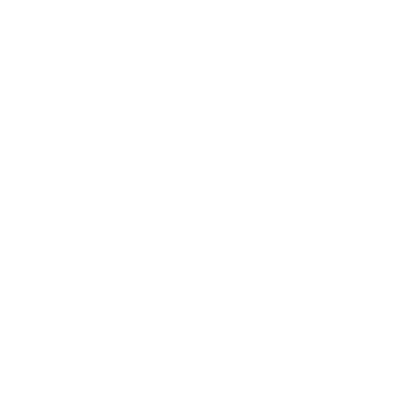

Nanomedicine has made significant impact to patients globally across disparate disease conditions ranging from schizophrenia and hypercholesterinemia to macular degeneration and various cancers; existing therapies include Doxil/Caelyx and Myocet for breast cancer, paliperidone palmitate for 3 monthly long-acting schizophrenia treatment, Visudyne for macular degeneration and Tricor for cholesterol management^1^,^2^,^3^. The growing pipeline of candidate nanomedicines continues to have options at various development stages, moving towards the ranks of clinically proven medicines. Nanotherapies often utilize injectable nanocarriers that deliver drug cargoes directly to the bloodstream; however, daily injections are not practical in many clinical scenarios, such as chronic conditions requiring self-administration over a protracted period. Such conditions render many nanomedicine approaches inappropriate, especially if disease is not restricted to targetable organs, creating a prerequisite for oral administration and requiring consideration of inter-related factors such as pharmacokinetics, patient adherence and pill burden. Many of these issues are exacerbated during paediatric administration where there is much less understanding^4^.

Daily oral administration of modern antiretroviral (ARV) therapy has transformed HIV from a fatal disease to a manageable chronic condition. However, access to ARVs is not universal and paediatric treatment formats still require improvement. The global burden of HIV continues to grow, with 2014 World Health Organization (WHO) statistics estimating 36.9 million people living with HIV, 2.6 million of which were children <15 years. At the end of 2014, an estimated 14.9 million people were receiving ARVs including only 32% of infected children. The ability to moderate progression of HIV to AIDS, in addition to ∼2 million new annual infections, places increasing demand on ARVs. The 2015 WHO guidelines^5^ recommend therapy initiation for everyone living with HIV at any CD4 cell count in addition to daily oral pre-exposure prophylaxis for at-risk populations, exacerbating supply pressure and stimulating therapy optimization strategies. Although attrition processing of large drug particles down to smaller particle sizes, using techniques such as high-pressure homogenization or nanomilling^2^,^6^, has led to many Food and Drug Administration-approved oral nanomedicines utilizing solid drug nanoparticles (SDNs), there are currently no oral ARV nanotherapies available. In addition, it is not clear what chemical and physical parameters (for example, size, surface chemistry, charge, charge density and polydispersity) of SDNs will successfully achieve clinical target performance and attrition processing is labour and time intensive. While many nanotechnologies are being explored for HIV, the achievement of large-scale production, under clinical manufacturing conditions and at low cost, is often not addressed.

In new cases of paediatric HIV infection (<3 years), a ritonavir (RTV)-boosted lopinavir (LPV) oral liquid formulation is WHO-recommended as a 4:1 LPV:RTV combination. Both LPV and RTV have low bioavailability with poor water solubility (1.92 and 1.26 mg l^−1^, respectively) and substrate affinity for transporters and metabolic enzymes^7^. Despite its well-known side effects, RTV is required as a pharmacoenhancer to ‘boost' the pharmacokinetics of other ARVs that are substrates for p-glycoprotein and/or cytochrome P450 3A4 (CYP3A4); a second drug, Cobicistat, is also approved as a booster for HIV therapies. For infants, the WHO-recommended twice-daily dose is 300 mg/75 mg LPV/RTV per m^2^ of body surface area, and a paediatric oral solution (80/20 mg ml^−1^) is available. Due to the poor water solubility of both drugs, the oral solution contains 42.4% (v/v) ethanol and 15.3% (w/v) propylene glycol. Administration may commence as early as 14 days after birth for prevention of mother-to-child transmission in HIV-positive mothers. Clearly, it would be preferential to avoid regular dosing of alcoholic mixtures, and viable strategies to remove RTV and/or alcohol are urgently required. Nanomedicine strategies may have a critical role to play in the formation of aqueous orally dosed paediatric therapies, but any strategy must allow rapid formation and evaluation of multiple SDN options and have cost and scalability at its core. We recently reported emulsion-templated freeze-drying (ETFD)^8^, a non-attrition approach to the production of aqueous re-dispersible SDNs, and its application to the ARV efavirenz^9^. The prototype nanomedicine exhibited higher pharmacokinetic exposure than a conventional preclinical formulation in rats after oral dosing.

Herein, we describe the extension of this strategy to the accelerated discovery and translation of LPV and LPV/RTV combination SDNs with high drug loading relative to excipients (≥50 wt%) through a unique iterative cycle of materials chemistry and pharmacology, utilizing a novel high-throughput ETFD screening process, with scale of production subsequently achieved by clinically compliant spray-drying. Reduction to lead candidates with optimized drug loading, larger pilot-scale production and stability testing has enabled formation of a candidate LPV nanomedicine for imminent human clinical trials (EudraCT number 2013-004913-41). By generating a formulation library with variability in size, surface charge and surface chemistry during screening stages, the identification of optimal nanoparticle properties as they relate to the pharmacology is possible. This process is highly translatable and offers the rapid production, evaluation and optimization of cost-effective orally dosed drug nanoparticles to address unmet clinical needs across various diseases.

## Results

### High-throughput screening of SDN candidates

**Figure 1:**
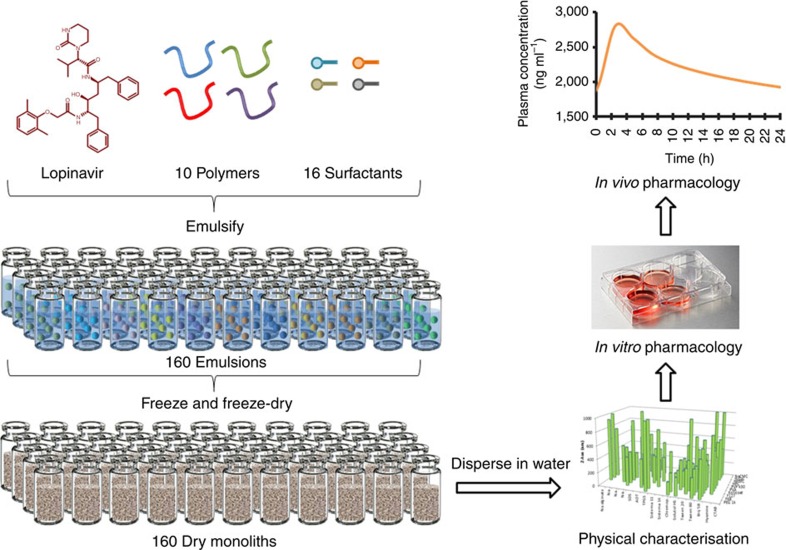
Schematic of the high-throughput manufacture of LPV SDN options. Aqueous solutions containing binary polymer and surfactant combinations act as the continuous phase to stabilize oil-in-water emulsions containing LPV within the internal phase. Rapid freezing, followed by freeze-drying, forms re-dispersible solid monoliths comprising water-soluble excipients and poorly water-soluble drug nanoparticles. Detailed physical and pharmacological characterization of SDN dispersions allows identification of SDNs with clinical potential.

**Figure 2:**
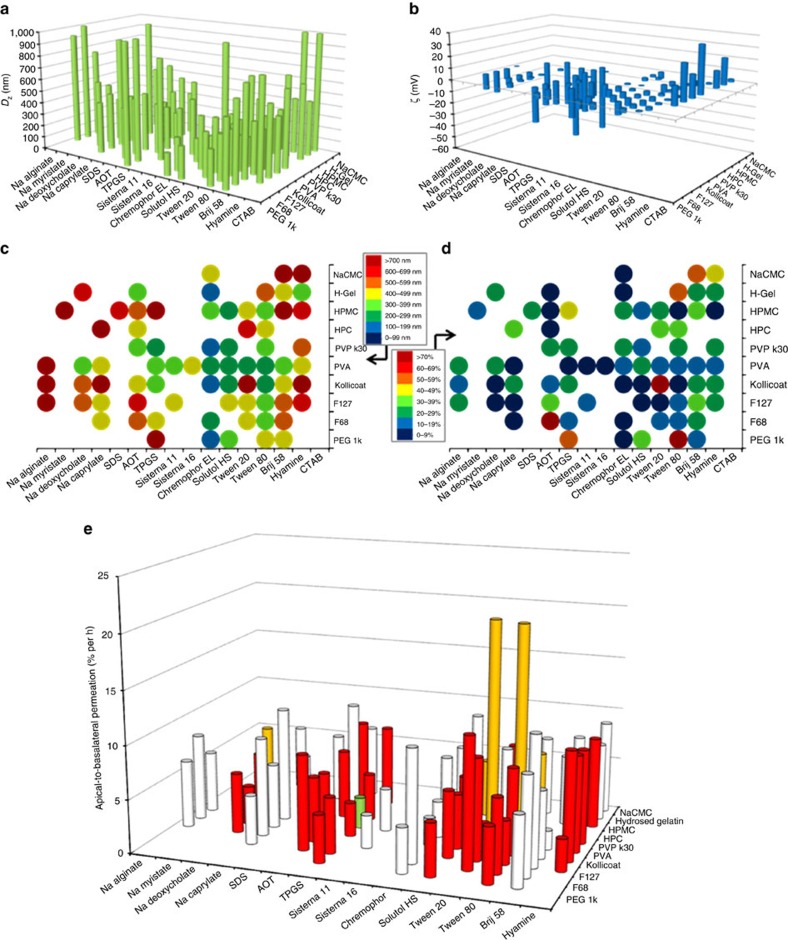
ETFD screening for LPV SDNs. Total solids per SDN 10 mg (10 wt% LPV, 60 wt% polymer and 30 wt% surfactant). Samples were analysed by DLS (25 °C) after dispersion in water at 1 mg ml^−1^ LPV. (**a**) *D*_*z*_ measurement of initial screen (blanks=sample did not fully disperse). (**b**) Corresponding *ζ*-values for successful SDNs. (**c**) *D*_*z*_ averages (n=3). (**d**) SDN reproducibility as % s.d. *D*_*z*_ (*n*=3). (**e**) Apical-to-basolateral permeation across Caco-2 cells. Excipient combinations able to form SDNs at 50 wt% LPV loading are highlighted in red; those forming 70 wt% LPV loadings are shown in amber; clinically progressed SDN is shown in green. (See Supplementary Table 1 and 3–7 for *D*_*z*_ and *ζ* values.)

SDN formation was initially studied using a miniaturized ETFD approach (Fig. 1); a 160-component library screening process was developed to determine candidate polymer and surfactant combinations, focusing first on single LPV SDNs with 10 wt% drug loading relative to water-soluble excipients. Oil-in-water emulsions were prepared from a chloroform (CHCl_3_)/water mixture with thorough emulsification utilizing externally focused ultrasonication to avoid cross-contamination. Excipient stabilizers with wide chemical and structural diversity were chosen from the Food and Drug Administration Center for Drug Evaluation and Research list of inactive ingredients^10^, ensuring pre-existent use in clinically approved medicines. Once sonicated, all volatile components were removed by freeze-drying to leave dry monolithic porous matrices of water-soluble excipients containing LPV SDNs. Monolith dispersion by addition of water (1 ml) led to the release of SDNs with stability derived from adsorption of the excipient polymers and surfactants. SDN hydrodynamic diameters (*D*_*z*_; Fig. 2a), polydispersity indices (PDI) and zeta potentials (*ζ*; Fig. 2b) were determined by dynamic light scattering (DLS). Unlike the serial formation of SDNs by attrition methods, the large number of SDNs successfully formed by ETFD required criteria to reduce the options to those most likely to succeed as scale-able nanomedicines including: (1) complete aqueous dispersion; (2) *D*_*z*_<1000, nm; (3) *D*_*z*_ s.d. between repeated measurement <10% (*n*=3; Fig. 2c,d); and (4) PDI<0.5. Although varying criteria could be chosen to narrow the options for further study, these were selected to ensure investigation of monomodal distributions of stable particles in the sub-micron range, generated after rapid dispersion; larger multi-micron particles are able to be readily generated using alternative methods.

Of the 160-component library, 74 candidates met these selection criteria and production was subsequently repeated to assess reproducibility (*n*=3) and confirm *ζ* values. Good reproducibility for each SDN candidate, with considerable *D*_*z*_ and *ζ* variability between library members was achieved (Supplementary Table 1). SDN formation was enhanced by non-ionic surfactants with correlation between *D*_*z*_ and surfactant use; greater *D*_*z*_ variation was observed with changing surfactant across each polymer option (Fig. 2c). Polyvinyl alcohol (PVA) and Kollicoat generated the most SDN candidates matching the selection criteria, presumably due to their surface active properties. To enable pharmacological evaluation, the 74 SDN candidates were each reproduced with integration of a tracer amount of ^3^H-radiolabelled LPV; this approach to the formation of radiolabelled SDNs is unique to ETFD to the best of the authors' knowledge.

### Pharmacological evaluation of SDNs comprising 10 wt% LPV

High-throughput screening of LPV apical-to-basolateral permeation (A>B; gut to blood) across Caco-2 cells was utilized for selection of leads with favourable pharmacology. An aqueous LPV control showed A>B permeation of 3.5% per hour. From the 74 LPV SDN candidates, most options showed comparable or greater A>B permeation (Fig. 2e). SDNs containing PVA with either Tween 20 or Tween 80 showed >5.5-fold greater transcellular permeation (20.5% per h). Importantly, a number of LPV SDN formulations also showed reduced permeation; SDNs comprising Kollicoat and Tween 20 showed a 1.4-fold lower rate (2.7% per hour). In addition, accumulation and cytotoxicity in Caco-2 cells was assessed (Supplementary Table 2). Only two formulations showed cellular accumulation ratios (CAR) higher than the LPV aqueous solution (CAR=35.3); sodium carboxymethyl cellulose (NaCMC) and Brij58 (CAR=36.6), and NaCMC and hyamine (CAR=39.6), indicating that augmented intracellular drug delivery was not driving increased permeation. No increase in overt cytotoxicity was observed. The considerable diversity in pharmacological behaviour of the SDN library, generated from this single compound, strongly underlines the need for a wide ranging study of the multiple chemical and physical variables that dominate SDN interactions with biological systems. Only through library generation and subsequent pharmacological testing can new SDN nanomedicine options be substantially de-risked to allow the investment of time and resources for further development.

### Optimizing SDN candidate drug loading and excipient ratios

Increasing drug content within SDN lead options is critical to cost-effective production and reduction of exposure to unnecessary excipients, particularly surfactants. Having established the potential for comparable or enhanced permeation through Caco-2 monolayers, and good reproducibility without compromising cytotoxicity, 49 candidates were chosen for progression.

A series of smaller libraries, restricted to the seven water-soluble surfactants and seven water-soluble polymers within the chosen 49 candidates, was generated with progressively increasing drug loading. ETFD libraries targeting ≤50 wt% LPV utilized the same oil-to-water phase ratio (1:4) and polymer:surfactant wt% ratio (40:10) as initial screens; libraries targeting up to 70 wt% LPV employed a 20:10 polymer:surfactant wt% ratio and successful SDN dispersions were characterized using DLS. Compared with the initial screen containing 10 wt% LPV, libraries comprising 50 wt% LPV (Supplementary Fig. 1; Supplementary Table 3) generated considerably fewer successful SDN options, with further reductions observed at a 70 wt% LPV loading (Supplementary Fig. 2; Supplementary Table 4). The polymers PVA, Kollicoat and hydroxypropyl methyl cellulose (HPMC) successfully generated SDNs at 50 wt% LPV; however, Kollicoat was unsuccessful at 70 wt%. PVA proved to be the most successful polymer at high drug/excipient ratios, with HPMC also showing considerable promise. The 49-component screen yielded six promising candidates at 70 wt% LPV. The inability of various excipients to generate SDNs at high drug content is possibly due to a lack of interaction with particles during formation under the ETFD process or an inability to provide sufficient surface nanoparticle coverage after dispersion. Each 49-component ETFD screen at 70 wt% LPV utilized 343 mg of drug, highlighting the importance of the initial 10 wt% screening process (160 mg LPV per 160-component library) to minimize drug use.

The polymer:surfactant ratios within the seven most promising 70 wt% LPV SDN options were varied from a 1:2 to a 5:1 wt ratio (*n*=2) to minimize surfactant (Supplementary Fig. 3; Supplementary Table 5). Resultant SDNs varied in *D*_*z*_ (566–984 nm) with near-neutral *ζ* values (−11 to 18 mV). Six 70 wt% LPV SDN formulations (Supplementary Table 6) were chosen for extended *in vitro* pharmacological evaluation due to their high drug loading and low surfactant content (5-10 wt%); these included binary mixtures of PVA:α-tocopherol polyethylene glycol succinate (TPGS), PVA:Tween 20 and PVA:sodium deoxycholate. LPV SDN options containing HPMC were not progressed at this stage due to the variability that was seen when using different batches of this excipient; with robustness as a key commercial need, a detailed investigation of this variability was not conducted.

### Combination 70 wt% LPV/RTV SDN formulations

ETFD is uniquely able to generate multiple-component SDNs by combining two or more hydrophobic compounds within the internal oil phase^11^,^12^; other approaches to combination nanoparticle approaches have utilized lipid nanocarrier approaches for subcutaneous injection^13^. This is of high clinical relevance for HIV due to the need for drug combinations. The ETFD conditions for 70 wt% LPV SDNs were utilized to generate LPV/RTV dual-component SDNs through substitution of a fraction of the LPV with RTV to provide a 56:14 w/w, matching the current 4:1 w/w clinical products while maintaining relative drug:excipient wt ratios. Recently, we showed RTV SDNs display augmented inhibition of CYP3A4, offering the potential for RTV dose reduction^14^. Therefore, combination SDNs with 10:1 and 40:1 wt ratios (LPV/RTV) were also generated. In general, very little variation in SDN formation and *D*_*z*_ was seen, but *ζ* values were observed to increase when substituting LPV with RTV (Supplementary Table 7).

### Monolith and nanodispersion characterization

The two lead SDN candidate formulations with good reproducibility, size and low surfactant content (70 wt% LPV and 56/14 wt% LPV/RTV, both with 20 wt% PVA and 10 wt% TPGS) were characterized in the solid state and as aqueous nanodispersions. Long-term stability is critical to translation and stable dosing dispersions within the pharmacy would considerably enhance convenience of use. LPV has at least four known crystal polymorphs and solvates with the thermodynamically stable form (Form 1) being preferred in conventional solid dosing^15^. Powder X-ray diffraction (p-XRD) of the ETFD lead SDNs showed fully amorphous materials (Supplementary Fig. 4a) with no observable crystallinity and no relevant variation in *D*_*z*_, *ζ* or PDI was observed during the 6-month study (Supplementary Fig. 4b–d). Aqueous SDN dispersions showed good stability for 10 h after dispersion; *D*_*z*_ increased slightly (600 nm to ∼750 nm) over 3 h with no further change over 8 h, and no substantial PDI or *ζ* variation (approximately −15 mV; Supplementary Fig. 4e-g). Good stability across a wide pH range 2.5–9.5 (Supplementary Fig. 4h) was seen with the expected *ζ* variation indicating predominantly steric stabilization. Similar investigations of LPV/RTV combination SDN monoliths and aqueous dispersions showed similar chemical and physical behaviour; amorphous material in the solid state, stable dispersions under varying pH and good stability when dispersed in water for 10 h (Supplementary Fig. 5).

### *In vitro* pharmacology of LPV and LPV/RTV 70 wt% SDNs

Gut absorption was evaluated using Caco-2 permeation of the aqueous SDN dispersions at various time points (1, 2, 3 and 4 h). In these extended studies, B>A and A>B permeation was compared with an aqueous LPV solution (Supplementary Table 8). At 1 h, the A>B transcellular permeation for each SDN comprising 70 wt% LPV ranged from 138.1 to 239.9 nmol, which is ∼2.5–4.8-fold higher than the measured 50.6 nmol for the LPV aqueous solution. Concomitantly, transcellular permeation in the B>A direction was similar to an aqueous solution for all but one SDN candidate at the 1 h time point (Supplementary Table 8). Over 4 h, one SDN formulation showed consistently greater A>B permeation compared with the aqueous LPV solution, with similar B>A permeation, suggesting bioavailability at least similar to conventional LPV as hoped for a bioequivalent paediatric format. This nanoformulation consisted of 70 wt% LPV, 20 wt% PVA and 10 wt% TPGS.

**Figure 3:**
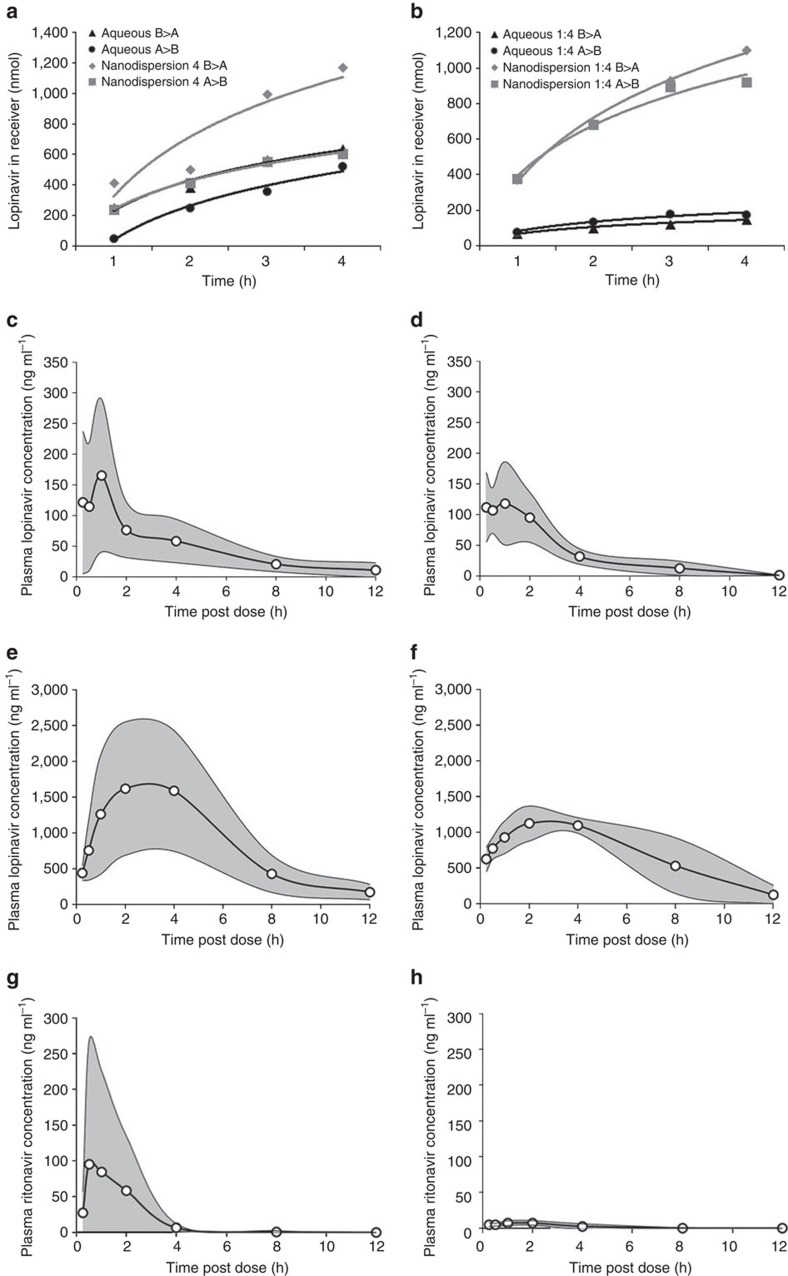
Summary pharmacology for lead candidate SDNs containing LPV or LPV and RTV. (**a**) Transcellular permeation of 70 wt% loaded lead LPV SDN across caco-2 cell monolayers. (**b**) Transcellular permeation of 70 wt% loaded LPV:RTV (4:1) SDN across caco-2 cell monolayers. (**c**) Pharmacokinetics of a conventional LPV preclinical formulation in rats after oral administration. (**d**) Pharmacokinetics of 50 wt% loaded spray-dried lead LPV SDN in rats after oral administration. (**e**) Pharmacokinetics of LPV from a conventional LPV:RTV (4:1) preclinical formulation in rats after oral administration. (**f**) Pharmacokinetics of LPV from a 50 wt% loaded spray-dried LPV:RTV (4:1) SDN in rats after oral administration. (**g**) Pharmacokinetics of RTV from a conventional LPV:RTV (4:1) preclinical formulation in rats after oral administration. (**h**) Pharmacokinetics of RTV from a 50 wt% loaded spray-dried LPV:RTV (4:1) SDN in rats after oral administration.

Drug permeation across Caco-2 monolayers from LPV/RTV combination SDNs was individually assessed. LPV permeation was greater than aqueous controls containing equivalent LPV/RTV for all combination SDNs tested across all time points (Supplementary Table 9): after 1 h, LPV permeation from combination SDNs was 380.9 nmol for a 4:1 ratio (control=77.6 nmol), 545.3 nmol from a 10:1 ratio (control=56.9 nmol) and 356.7 nmol from a 40:1 ratio (control=44.1 nmol). After 4 h, LPV permeation from combination SDNs was 922.5 nmol from a 4:1 ratio (control=176.3 nmol), 872.5 nmol from a 10:1 ratio (control=124.6 nmol) and 854.5 nmol from a 40:1 ratio (control=79.2 nmol). RTV permeation from combination SDNs with a 4:1 or 10:1 LPV/RTV ratio was similar to the respective aqueous controls and greater for combination SDNs with a 40:1 ratio. Based upon the Caco-2 permeation data (Supplementary Table 8; Fig. 3a,b) for the lead candidates, and focussed on acceleration towards future translation, progression of only one single and one combination SDN formulation to pilot-scale clinical manufacture was conducted. LPV SDNs allow study of variation in RTV (dosed using conventional 50 mg tablets) levels and the potential for manipulation of drug ratios to maintain bioequivalence of LPV, rather than the single study of one fixed combination SDN; LPV SDNs were therefore selected for progression to large-scale manufacture.

The use of Caco-2 monolayers for relatively rapid evaluation of gut permeation has been reported by many groups, but recent reports of co-culture and triple-culture models that allow the incorporation of mucous within the *in vitro* models have sought to extend the relevance of such studies^16^. Permeation of the selected LPV SDNs through two additional co-culture models and one triple-culture model was studied to determine gross variation between models.

Permeation of 50 wt% loaded spray-dried lead LPV SDN across the cellular layer of triple-culture model was assessed to determine the influence of mucous and M cells on SDN permeation (Supplementary Table 10). The presence of HT29-MTX-cells in the monolayer resulted in a significant decrease in SDN permeation at all time points assessed (*P*<0.05), presumably due to the inhibiting presence of a more developed mucous layer covering the apical surface of the cells. Compared with the data from Caco-2 monolayers, double-culture monolayers containing both Caco-2 and M cells did not alter the cellular permeation of the LPV SDN at 1 h (*P*=0.24), 2 h (*P*=0.18), 3 h (*P*=0.065) or 4 h (*P*=0.093).

Cellular toxicity of aqueous LPV, 50 wt% loaded spray-dried lead LPV SDN, PVA and TPGS were assessed over 24 h in Caco-2 cells (Supplementary Fig. 6). The aqueous LPV, 50 wt% loaded spray-dried lead LPV SDN, and PVA were not toxic at any concentration tested, therefore, had CC_50_ above 50 μM. The TPGS caused toxicity at the higher concentrations assessed with a CC_50_ of 201 μM.

### Translation of ETFD SDNs to pilot-scale clinical manufacture

ETFD library screening identified SDN options with potential as orally dosed HIV nanomedicines. The removal of alcohol from current paediatric treatments and the opportunity to investigate the impact of RTV boosting of SDNs led to cost-effective large-scale production studies. Several challenges were identified including low solid content within ETFD screening (2% w/v). Spray-drying of emulsions containing volatile organic solvents is not commonplace, but solutions of dichloromethane (CH_2_Cl_2_) have been previously spray-dried at scale^17^. We aimed to optimize processing conditions for large-scale LPV SDN manufacture while translating to emulsion spray-drying (ESD) and maintaining *D*_*z*_, PDI, *ζ* and pharmacological behaviour for the candidate SDNs. Inexpensive laboratory grade PVA (80% hydrolysed, molecular weight (MW)=9,500 g mol^−1^) was replaced with pharmaceutical grade PVA (grade 4–88, MW=57–77,000 g mol^−1^) and the heat stability of LPV was investigated to determine temperature limits for spray-drying. In addition, CHCl_3_ was replaced with CH_2_Cl_2_ as the emulsion internal phase. The International Conference on Harmonisation Steering Committee categorizes CHCl_3_ and CH_2_Cl_2_ as class 2 solvents. However, the residual solvent limits for CH_2_Cl_2_ are 600 p.p.m. (whereas CHCl_3_ limit=60 p.p.m.) allowing greater scope for reaching acceptable limits. The boiling point of CHCl_3_ (61.2 °C) and low volatility during small-scale sonication makes it ideal for ETFD, but the lower boiling point of CH_2_Cl_2_ (39.6 °C) presents a greater opportunity for efficient removal during aerosolisation within the heated carrier gases of a spray-dryer. High-performance liquid chromatography (HPLC) analysis of LPV solutions in CH_2_Cl_2_ showed no degradation over 24 h and similar analysis of spray-dried emulsion samples (10 ml) showed 99.6% LPV recovery when using inlet temperatures up to 220 °C (outlet=114 °C).

SDN formation by ESD was conducted by processing the emulsions identified using ETFD (70 wt% LPV/20 wt% PVA/10 wt% TPGS; 1:9 oil:water phase ratio; 2 wt% total solids) using a Buchi benchtop spray-dryer. Initial experiments failed to form SDNs at 70 wt% LPV and stepwise reduction of LPV to 50 wt% yielded SDNs from readily water-dispersible powders with very similar results to those formed during ETFD (*D*_*z*_=625±18 nm, PDI=0.47±0.03, *D*_*n*_=337±49 nm). Optimization of solids content within feedstocks was also conducted in a stepwise fashion, increasing from 2% w/v to 8 w/v % by simple removal of water from the emulsion. Feedstock concentrations are a critical determinant of achieving cost-effective scale when spray-drying. Variation in probe sonication parameters made little impact on emulsion or SDN formation, but spray-dry conditions were adjusted to increase flow through the spray-dryer, resulting in an inlet temperature of 140 °C and subsequent outlet of 70 °C. Optimized emulsions comprised CH_2_Cl_2_ (2 ml, 200 mg ml^−1^ LPV) and an aqueous PVA/TPGS solution (6.4 ml and 1.6 ml respectively, 50 mg ml^−1^) in a 1:4 oil/water phase ratio with an overall 8 w/v % solid loading (50 wt% drug loading). Spray-drying yielded readily water-dispersible solid powders releasing SDNs with *D*_*z*_=393±6 nm, PDI=0.28±0.03, *D*_*n*_= 272±12 nm. Substitution of 20 wt% of LPV with RTV, to make combination LPV/RTV SDNs, successfully generated powders that dispersed to give SDNs of *D*_*z*_=441±16 nm, PDI=0.24±0.01, *D*_*n*_=290±84 nm.

Emulsion volumes were increased to 300 ml with excellent reproducibility of LPV SDNs (no RTV) using the benchtop spray-dryer and subsequent progression to semi-pilot-scale (Niro Mobile Minor) where a series of 20 emulsions were successively fed through the spray-dryer over 4 h leading to 306 g of SDN-containing powder (64% mass recovery). After vacuum drying under ambient conditions, LPV content was determined by HPLC as 53 wt% with no measureable impurities. This material was shown to be amorphous via p-XRD, yielding dispersed SDNs on dispersion into water (*D*_*z*_=404 nm, PDI=0.28 and *D*_*n*_=256 nm, *ζ*=−1.1±0.09 mV). The stability of the amorphous SDNs was studied under controlled conditions over 12 months at elevated temperature (40 °C and 75% relative humidity) with no indication of crystallinity or degradation over this period (Supplementary Table 11); in addition, samples stored under ambient conditions for 14 months also showed no observable onset of crystallinity via p-XRD analysis (Supplementary Fig. 7). Gas chromatography headspace analysis confirmed residual CH_2_Cl_2_ concentrations within International Conference on Harmonisation limits.

### Physiologically based pharmacokineticsimulations

Using this physiologically based pharmacokinetic (PBPK) model (see Methods), Caco-2 cell data were used to predict the pharmacokinetics of SDN (with highest apparent permeability from the screen) and the traditional formulation of LPV in a virtual cohort of 100 patients receiving standard doses of 400/100 mg twice daily (Supplementary Fig. 8). The simulation predicted a higher *C*_max_ (maximum concentration achieved in the plasma; 11.915±3.631 versus 7.449±2.916 mg l^−1^) and higher *C*_min_ (minimum concentration in the plasma at the time of second dose; 6.045±2.736 versus 4.023±1.981 mg l^−1^) for the SDN versus the conventional formulation.

### *In vivo* pharmacokinetic evaluation of spray-dried LPV SDNs

The pharmacokinetics of the orally dosed LPV SDNs and LPV/RTV combination SDNs was studied *in vivo* by WuXiAppTec (http://www.wuxiapptec.com/) in compliance with their internal policies. Adult male Sprague-Dawley rats were dosed with conventional preclinical formulations of LPV or LPV/RTV and equivalent aqueous SDN dispersions. Blood samples were taken over 12 h and plasma concentrations of LPV and/or RTV were determined using mass spectrometry (Fig. 3c–h). LPV exposure after dosing of single-component SDNs or conventional preclinical formulation was not significantly different despite the absence of organic liquids to aid dissolution: average *C*_max_ LPV=187 ng ml^−1^ and LPV SDN=155 ng ml^−1^ (*P*=0.60); average area under the curve (AUC) LPV=579.4 ng ml^−1^ h^−1^ and LPV SDN=434 ng ml^−1^ h^−1^ (*P*=0.37); *T*_max_ LPV=0.6 h and LPV SDN=1 h (*P*=0.68).

Comparison of LPV plasma concentrations for rats orally dosed with LPV/RTV (4:1) combination SDNs (Fig. 3f) and equivalent preclinical formulations (Fig. 3e) showed lower pharmacokinetic exposure to LPV after administration of the combination SDN, but these differences did not reach statistical significance: average LPV/RTV *C*_max_=1,738 ng ml^−1^ and LPV/RTV SDN=1,194 ng ml^−1^ (*P*=0.23); average LPV/RTV AUC=10,540 ng ml^−1^ h^−1^ and LPV/RTV SDN=8,423 ng ml^−1^ h^−1^ (*P*=0.33). The similarity of LPV plasma concentrations from combination SDNs is remarkable with relatively poor RTV absorption with average AUC calculated as 213 ng ml^−1^ h^−1^ from the conventional preclinical formulation and 28 ng ml^−1^ h^−1^ for combination SDNs (*P*<0.05).

The observation that plasma RTV concentrations were lower in the LPV/RTV SDN despite equivalent LPV exposure is counterintuitive given that RTV is needed to attain therapeutic LPV concentrations. To clarify this, the concentrations of LPV and RTV were investigated in hepatic tissue homogenates. Interestingly, LPV hepatic concentrations were 15.2±13.7 ng g^−1^ versus 1.5±3.4 for the conventional versus the LPV/RTV SDN formulation, respectively (*P*=0.048). Conversely, RTV hepatic concentrations were equivalent between the conventional and the LPV/RTV SDN formulation at 10.8±7.5 versus 6.6±6.7 ng g^−1^, respectively (*P*=0.41). Therefore, a change in LPV route of entry into the systemic circulation (for example, via the lymphatics) may reduce entry via the hepatic portal vein directly to the liver, meaning a lower hepatic LPV concentration and a reduction in RTV concentrations necessary for pharmacoenhancement. However, further experimentation would be needed to confirm this mechanism.

Due to all of these observations, the single-component LPV SDN formulation was progressed to enable more flexibility for RTV dosing within subsequent clinical trials (that is, the dose of RTV needed for optimal pharmacoenhancement can be titrated in humans using conventional RTV tablets, which would not be possible using a fixed-dose combination LPV/RTV SDN). For clarity, these initial human trials will considerably inform the drug ratios within candidate combination LPV/RTV SDNs and the development of target ethanol-free paediatric therapies. It is notable that a general reduction of variability was observed in both *in vivo* studies, potentially offering greater predictability if this behaviour is replicated in future human studies.

### Storage characterization of ESD LPV SDN formulations

ESD powders containing LPV SDNs were filled into Size 0 Vcaps Plus HPMC capsules (Capsugel) to a dose of 150 mg of LPV per capsule, placed in 50 ml high-density polyethylene bottles and subjected to accelerated storage stability testing for 6 months at 40 °C/75% RH and for 12 months at 25 °C/60% RH to establish long-term stability before submission for approval of first-in-human clinical studies. In both cases, analysis by HPLC showed related substances below limits defined within the US Pharmacopeial Convention specification^18^, confirming viability as a candidate clinical nanomedicine (Supplementary Tables 11 and 12).

## Discussion

The treatment of HIV requires oral dosing as the only patient-acceptable daily administration option. Chronic oral dosing has significant complications that arise from the high pill burden experienced by many patients across populations with varying conditions leading to subsequent non-adherence to regimens. Recent evaluation of HIV patient groups have shown a willingness to switch to nanomedicine alternatives if benefits can be shown^19^ and research efforts focused on the development of long-acting subcutaneous and intramuscular injections, using SDNs produced via milling approaches, may address many oral dosing challenges^20^. However, until long-acting combination therapies are available orally dosed ARVs will continue to be mainstay therapies.

Orally dosed SDNs have been successfully deployed for many disease conditions and the use of appropriate SDNs for HIV is a natural progression. Circulating SDNs are not expected to be present after gut absorption and, in our current study, the SDN benefits are likely derived from benefits in the intestine without reliance on added solvents; as suggested by their stability at varying pH (Supplementary Figs 4 and 5), the SDNs are expected to remain as intact nanoparticles in the intestine, thereby presenting the observed benefits. The pharmacokinetic (PK) profiles in rodents are very encouraging and offer the ability to provide a novel aqueous dosing format at identical dosing levels, but without the use of ethanol and with the storage benefits of a re-dispersible solid, in contrast to the current liquid product containing volatile solvents.

Although the data are highly promising, the rat studies presented here do not necessarily correlate directly to human adult, or paediatric, PK behaviour and the full opportunities for SDNs of ARVs will only be truly seen after oral dosing to humans. Although the single-component LPV SDNs have been progressed to clinical manufacturing scale, opportunities may also be available for the combination LPV/RTV SDNs. Indeed, the maintenance of *C*_min_ with reduced LPV *C*_max_ and reduced overall exposure to RTV observed in rat studies may be important in the context of concentration-dependent and chronic-exposure toxicities for these drugs^21^. It is also worth noting that early screening identified formulations with the potential for augmented exposure but for the purposes of this project, and simplified regulatory acceptance, a single-component LPV paediatric formulation with the potential for bioequivalence in an entirely aqueous format is being progressed to clinical evaluation (EudraCT number 2013-004913-41) due to the pressing clinical need for ethanol-free therapy options.

Preliminary ESD studies to establish commercially relevant manufacture of combination SDNs were successful through simple substitution of 20 wt% of LPV with RTV. Currently, the clinical LPV/RTV solid-dose formulation (Kaletra) is processed by melt extrusion and the paediatric liquid formulation has limited shelf-life of 2 months (at 25 °C) if not refrigerated, making this option non-ideal for use in sub-Saharan Africa^22^.

The increasing demand for ARV manufacture is a growing concern and the data generated for the SDNs studied here do not appear to offer routes to decrease oral dose. However, the nanomedicine options presented are cheap to produce in a simple manufacturing process, and have a demonstrated 6-month shelf-life at elevated temperature that should allow a reduction in distribution costs with a predicted decrease in waste.

**Figure 4:**
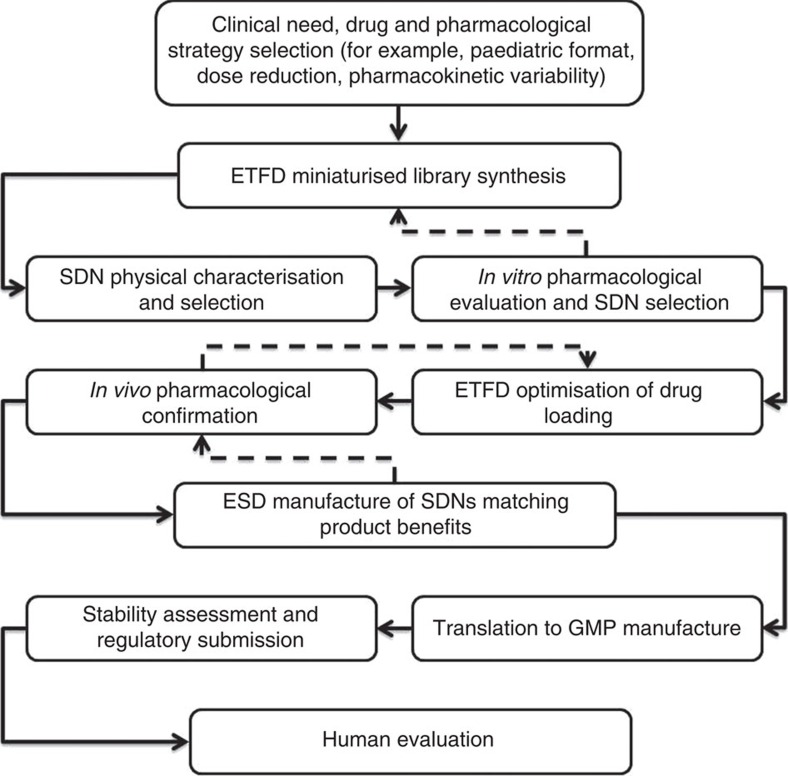
Candidate SDN progression strategy. Accelerated approach taken to address unmet clinical needs with oral nanomedicines, from library screening through to preclinical confirmation of benefits, formulation optimization and regulatory translation. Solid lines show the progression of candidates through the evaluation process, which incorporates preclinical pharmacology into key selection points. Areas of intense iteration between materials synthesis and pharmacological validation are shown by dashed lines.

The strategy described throughout this study is explicitly illustrated in Fig. 4, and has a number of key benefits. First, it is clear that the ETFD screening strategy allows for a large number of SDN nanoparticle options to be generated and rapidly assessed for chemical and physical properties. This is unique within our accelerated strategy as hundreds of SDN options may be generated within days, allowing for multiple options to be removed from the evaluation process, in contrast to the relatively small number of SDN options that could be studied in an identical period using homogenization or nano-milling. Second, it is also clear that the libraries of SDNs behave very differently pharmacologically and it would be impossible to predict which initial combination of excipients would provide the appropriate size, surface charge, stability and permeability to provide the best options for further study; in essence, by generating and testing the large number of options, any discipline prejudice is removed and the SDNs select themselves for progression. This is important as a conventional serial approach is unlikely to identify optimum materials from limited data sets.

The number of potential options identified does present a series of issues as seen in Fig. 2e; options to enhance bioavailability were present within the initial gut-model permeation evaluation but the target within this study was the generation of bioequivalent options that would presumably avoid potential complications that can arise from fundamentally new absorption and bio-distribution as seen with some previous clinically accepted nanomedicines^23^,^24^. In addition, the screening process allowed multiple optimization steps to be simultaneously attempted to increase drug loading relative to excipients. If conducted in a stepwise and serial manner, this parallel approach would have taken considerably longer to achieve very high loadings. Finally, the progression to clinical manufacture through ESD has created a commercially viable nanotherapy, identified through library-based screening utilizing just 1 mg per library sample and translated to near-kg scale production ready for human trials. This has considerable implications for future SDN-based nanomedicine development programmes as the timescale of initial laboratory screening to human evaluation (<4 years) should provide a pathway for acceleration of a range of new therapeutic opportunities. At the time of manuscript preparation, the authors have received UK Medicines and Healthcare products Regulatory Agency approval for human bioequivalence trials and will be commencing dosing to healthy volunteers in the near future.

## Methods

Material details and some routine experimental information are available in the Supplementary Methods.

### Preparation of emulsion-templated freeze-dried monoliths

Stock solutions of LPV or 4:1 LPV/RTV (10, 50 or 70 mg ml^−1^ in chloroform), polymer (P; 22.5 mg ml^−1^ in water) and surfactant (S; 22.5 mg ml^−1^ in water) were prepared using standard techniques. The three stock solutions were added to a sample tube, along with additional water (W) in the following API:P:S:W (μl) ratios: 100:267:133 (10 wt% API, 60 wt% P and 30 wt% S), 100:178:45:177 (50 wt% API, 40 wt% P and 10 wt% S) and 100:90:45:265 (70 wt% API, 20 wt% P and 10 wt% S) to form a total solid mass (10 mg) in a 1:4 chloroform/water mixture. The samples were emulsified using either a Hielscher UP400S ultrasonic processor equipped with H3 probe at 50% (Wattage) output intensity for 7 s or using a Covaris S2x acoustic homogenization system for 30 s with a duty cycle of 20, an intensity of 10 and 500 cycles/burst in frequency sweeping mode. Samples were cryogenically frozen immediately and lyophilized using a Virtis benchtop K freeze-dryer for 48 h. If required, ^1^H-labeled LPV was added to the chloroform stock solution to generate radioactive SDNs.

### Preparation of emulsion spray-dried powders

(Note: the following describes 300 ml scale processing). API stock solutions of LPV or 4:1 LPV/RTV (200 mg ml^−1^ in dichloromethane), polymer (P) PVA (grade 4–88, MW 57–77,000; 50 mg ml^−1^ in water) and surfactant (S) Kolliphor TPGS (50 mg ml^−1^ in water) were prepared using standard techniques. Three stock solutions were mixed in the API:P:S ratio 60:192:48 (ml) in a 1:4 dichloromethane to water mixture. Emulsified was conducted using a Hielscher UP400S ultrasonic processor equipped with H14 Probe at 100% output (140 W) for 300 s, with immediate spray-drying using a benchtop spray-dryer (BUCHI Mini-290) with an air-atomizing nozzle and compressed air as the drying gas. Spray-drying process conditions: 7 ml min^−1^ solution flow rate; 65 °C outlet temperature; and 110 °C inlet temperature. Resultant powders were dried under vacuum for 48 h.

### Cytotoxicity studies

Cytotoxicity was conducted as previously described^9^. In brief, caco-2 cells were seeded at 2.5 × 10^4^ cells 100 μl^−1^ into a 96-well plate and incubated for 24 h. Media were replaced with media containing 0.1 × 10^−6^, 1 × 10^−6^, 10 × 10^−6^, 100 × 10^−6^, 500 × 10^−6^ or 1,000 × 10^−6^ M LPV SDN or LPV aqueous solution (<1% dimethylsulphoxide) and incubated for a further 24 h. Subsequently, CellTiter-Glo Reagent (100 μl) was added and luminescence was measured after 10 min using a Tecan Genios plate reader (Tecan; Austria). In addition, the 50 wt% loaded spray-dried lead LPV SDN toxicity was assessed in Caco-2 cells. Media were replaced with media containing 0.1 × 10^−6^, 0.2 × 10^−6^, 0.39 × 10^−6^, 0.78 × 10^−6^, 1.56 × 10^−6^, 3.13 × 10^−6^, 6.25 × 10^−6^, 12.5 × 10^−6^, 25 × 10^−6^ or 50 × 10^−6^ M LPV as either aqueous solution or in SDN form (<1% dimethylsulphoxide) and incubated for a further 24 h. Excipients present in the SDN (PVA and TPGS) were also assessed using the concentrations ranging from 500 × 10^−6^ to 1 × 10^−6^ M. Analysis was performed as described above.

### Cellular accumulation studies

Cellular accumulation was determined as previously described^9^. In brief, caco-2 cells were seeded at 5 × 10^6^ per well and after 24 h, media was replaced with HBSS containing 10 × 10^−6^ M (final concentration) LPV in either solution or SDN form with 0.1 μCi of ^3^H LPV as a tracer. After 60 min, an extracellular sample (100 μl) was placed into a scintillation vial. Cells were lysed in water (500 μl) and plates were incubated for 24 h at −20 °C. Ultima gold liquid scintillation cocktail (4 ml) was added to intracellular and extracellular samples and radioactivity detected using a Perkin Elmer 3100TS scintillation counter. CARs were calculated as the ratio of intracellular/extracellular concentrations.

### Transcellular permeability studies

Transcellular permeability assays utilized Caco-2 cells propagated to a monolayer over 21 days, yielding transepithelial electrical resistance values of >1,300 Ω. LPV solution or LPV SDNs (10 × 10^−6^ M; including 0.1 μCi ^3^H LPV) was added to the donor chamber of four wells and a sample was drawn (and replaced) from the receiver compartment over 4 h. In addition, the transcellular permeability of 50 wt% loaded spray-dried lead LPV SDN was assessed through single-, dual- and triple-culture monolayer models, as described previously^16^. Mannitol permeability was used to determine monolayer integrity as described above. Antipyrene was assessed as a high permeability control compound that is largely unaffected by the presence of a mucous layer or M cells.For the PBPK modelling, virtual patients were generated using a population physiology model^25^. Oral absorption was simulated using a compartmental absorption and transit model and the volume of distribution was simulated using the Poulin and Theil method^26^,^27^,^28^,^29^. The PBPK model was designed using Simbiology v.4.3.1, a product of Matlab v.8.2 (MathWorks, Natick, MA, 2013). *In vitro* data describing physicochemical properties, absorption, distribution, metabolism and elimination of LPV and RTV were determined experimentally or obtained from the literature. The PK of LPV/RTV was simulated in 100 virtual individuals treated with 400/100 mg bid. The impact of SDN on the absorption and pharmacokinetics of LPV was simulated considering the highest and lowest Papp obtained for the apparent permeability assay in Caco-2 cells.

### *In vivo* studies

*In vivo* analyses were conducted using adult male Sprague-Dawly rats (200–300 g; five animals per group). Animals were surgically implanted with a catheter in a carotid artery using polyethylene tubing heparin (50I U ml^−1^)/glucose (50%) used as the lumen lock solution and allowed to recover for 3–5 days before the experiment. Doses were prepared on the day of the experiment and administered via oral gavage. Blood samples (0.25 ml) were collected via the catheter at the presented time points post dose. Plasma was prepared within 30 min of being drawn and stored at −70 °C until analysis of LPV and RTV (where relevant) by liquid chromatography–tandem mass spectrometry. SDN formulations were dispersed in water before administration, whereas conventional formulations consisted of 95% propylene glycol with 5% ethanol (as used in preclinical development by the originator)^30^.

### Data availability

Data supporting the findings of this study are available within the article and its Supplementary Information files and from the corresponding author upon reasonable request.

## Additional information

**How to cite this article:** Giardiello, M. *et al*. Accelerated oral nanomedicine discovery from miniaturized screening to clinical production exemplified by paediatric HIV nanotherapies. *Nat. Commun.* **7,** 13184 doi: 10.1038/ncomms13184 (2016).

## Supplementary Material

Supplementary Information Supplementary Figures 1-8, Supplementary Tables 1-12 and Supplementary Methods.
