# Supplementary material for: Accelerated oral nanomedicine discovery from miniaturized screening to clinical production exemplified by paediatric HIV nanotherapies
Source: Nat Commun. 2016 Oct 21;7:13184. doi: 10.1038/ncomms13184 (PMC5078733; doi:10.1038/ncomms13184)
Supplement: Supplementary Information — Supplementary Figures 1-8, Supplementary Tables 1-12 and Supplementary Methods. [file ncomms13184-s1.pdf]

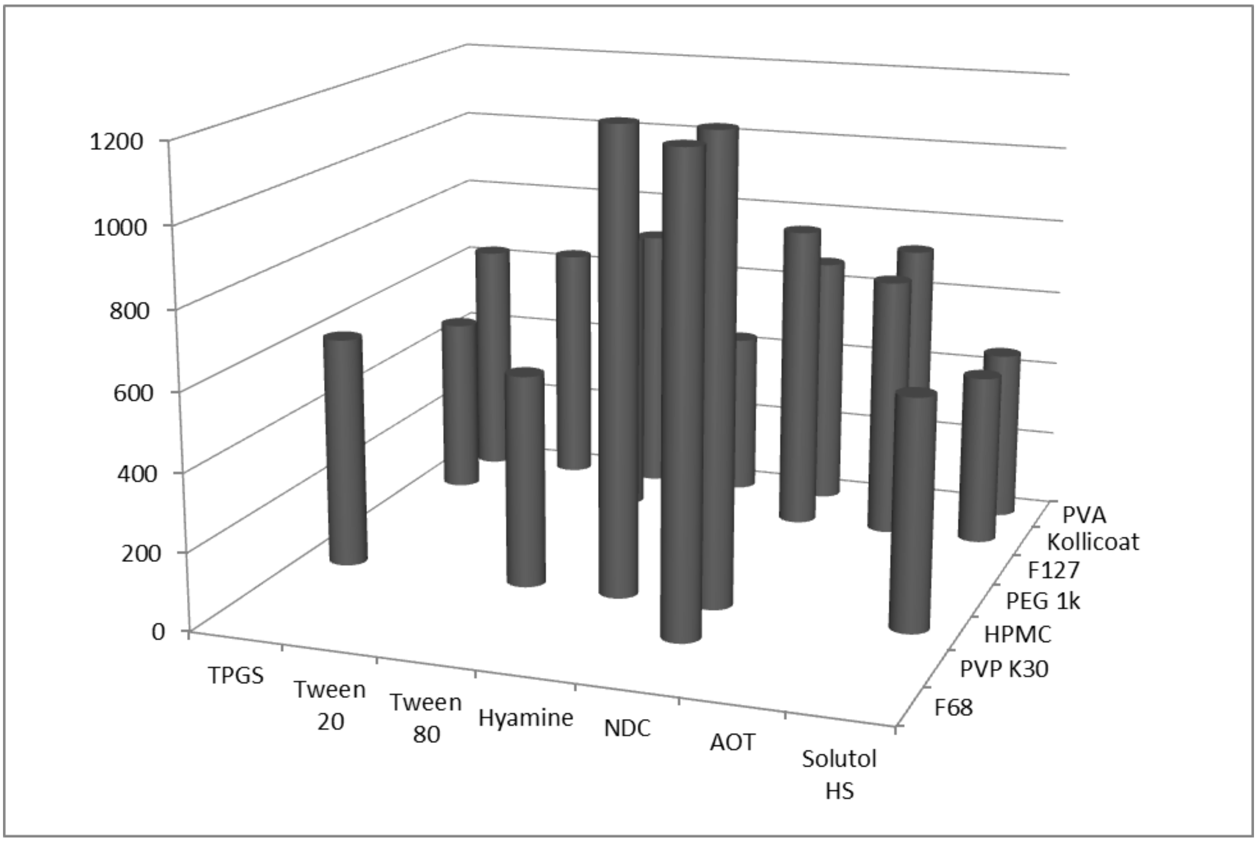

Supplementary Figure 1: D_z for screens utilising 50% LPV, 40% Polymer, 10% surfactant. Measurements determined by DLS, samples dispersed at 1 mg mL^{-1} in water at 25 ^oC

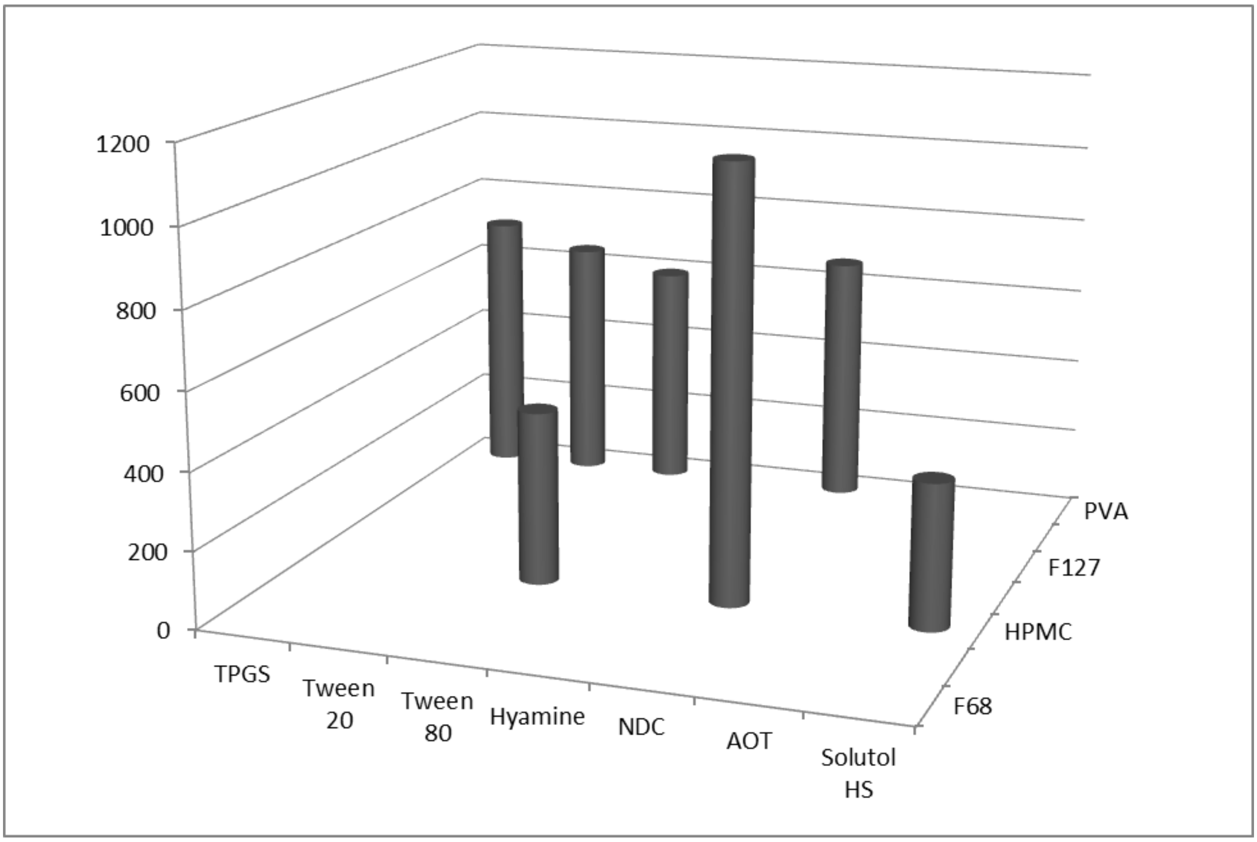

Supplementary Figure 2: D_z for screens utilising 70% LPV, 20% Polymer, 10% surfactant. Measurement DLS, samples dispersed at 1 mg mL^{-1} in water at 25 ^oC

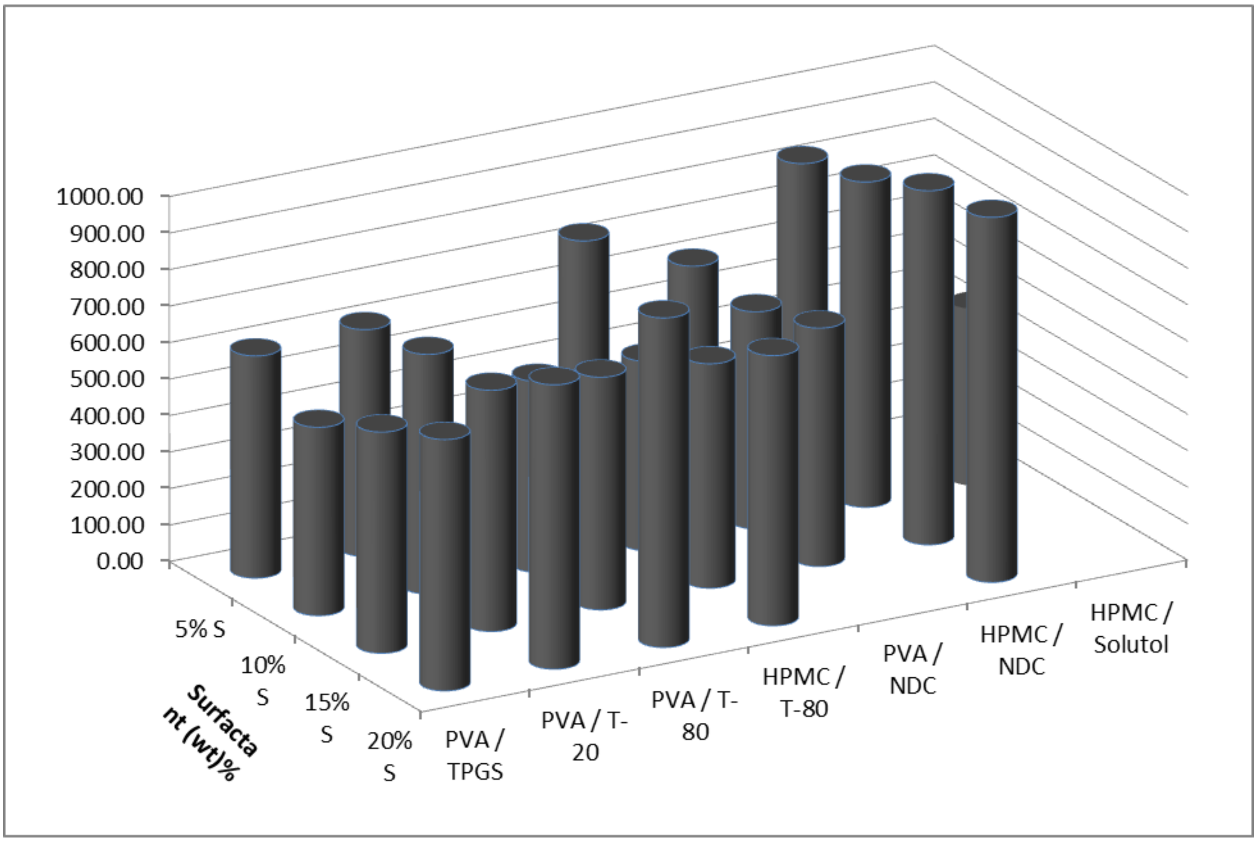

Supplementary Figure 3: D_z for variation of surfactant/polymer ratio; 70% LPV constant. Measurements determined by DLS, samples dispersed at 1 mg mL^{-1} in water, 25 ^{o}C

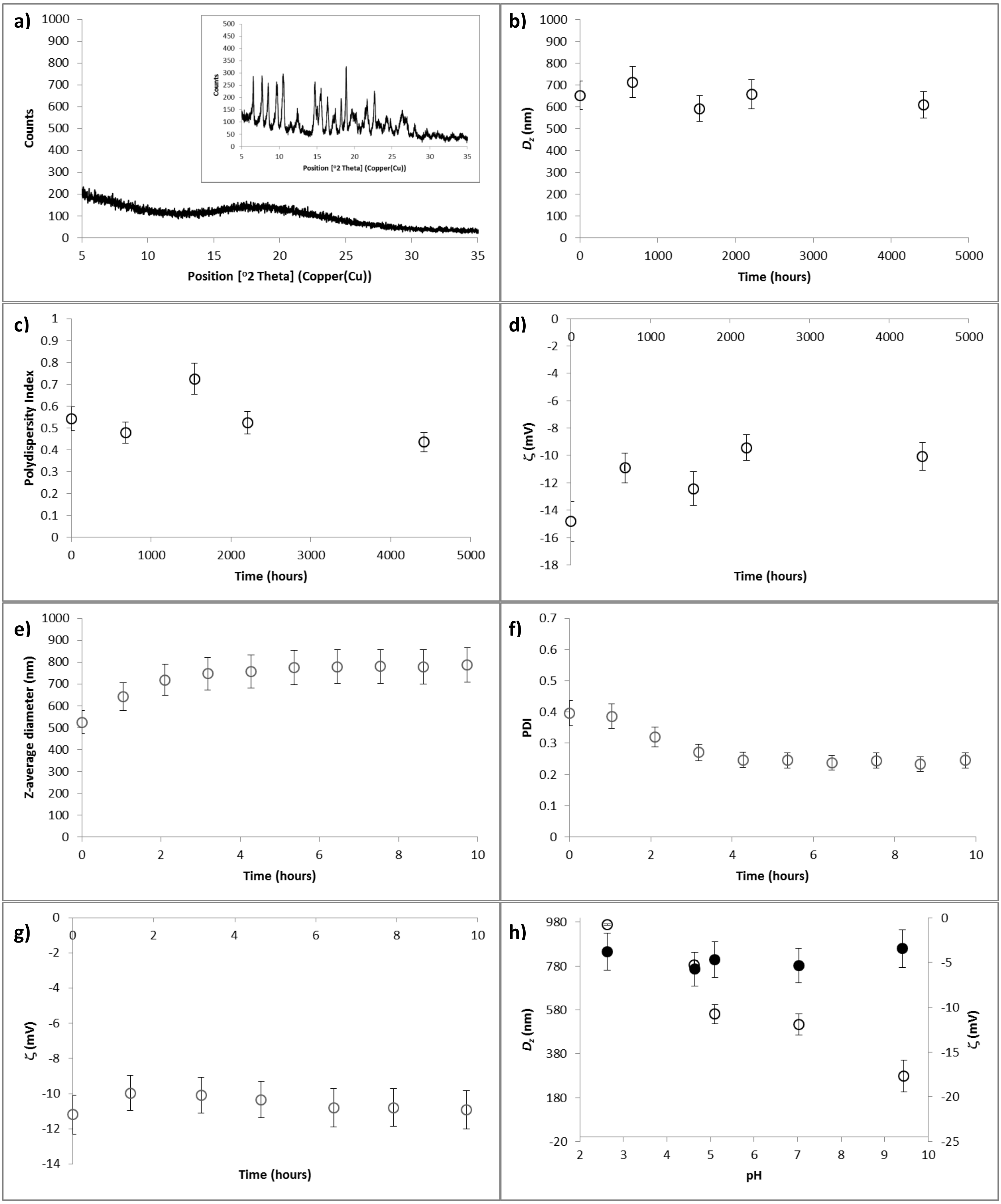

Supplementary Figure 4: Physical characteristics of solid state and dispersed ETFD lead SDN candidate (70 wt% LPV, 20 wt% PVA, 10 wt% TPGS. a) p-XRD of the ETFD monolith recorded directly following freeze drying (insert shows non-processed LPV powder). b) D_z, c) PDI and d) ζ monitoring of stored ETFD monoliths after redispersion; several ETFD monoliths were prepared and stored under ambient conditions. Individual samples were redispersed approximately each month (1 mg mL^{-1} in water) and the resulting nanodispersion was characterised by DLS. e) D_z, f) PDI and g) ζ monitoring of ETFD aqueous dispersions; ETFD monoliths were dispersed in water (1 mg mL^{-1}) and monitored via DLS for 10 hours (stationary samples). h) D_z (filled circles) and ζ (open circles) of ETFD dispersions (1 mg mL^{-1} in water) at varied pH. In all cases, samples were studied at 25 °C and fixed error bars are shown derived from instrumental accuracy. (10% error).

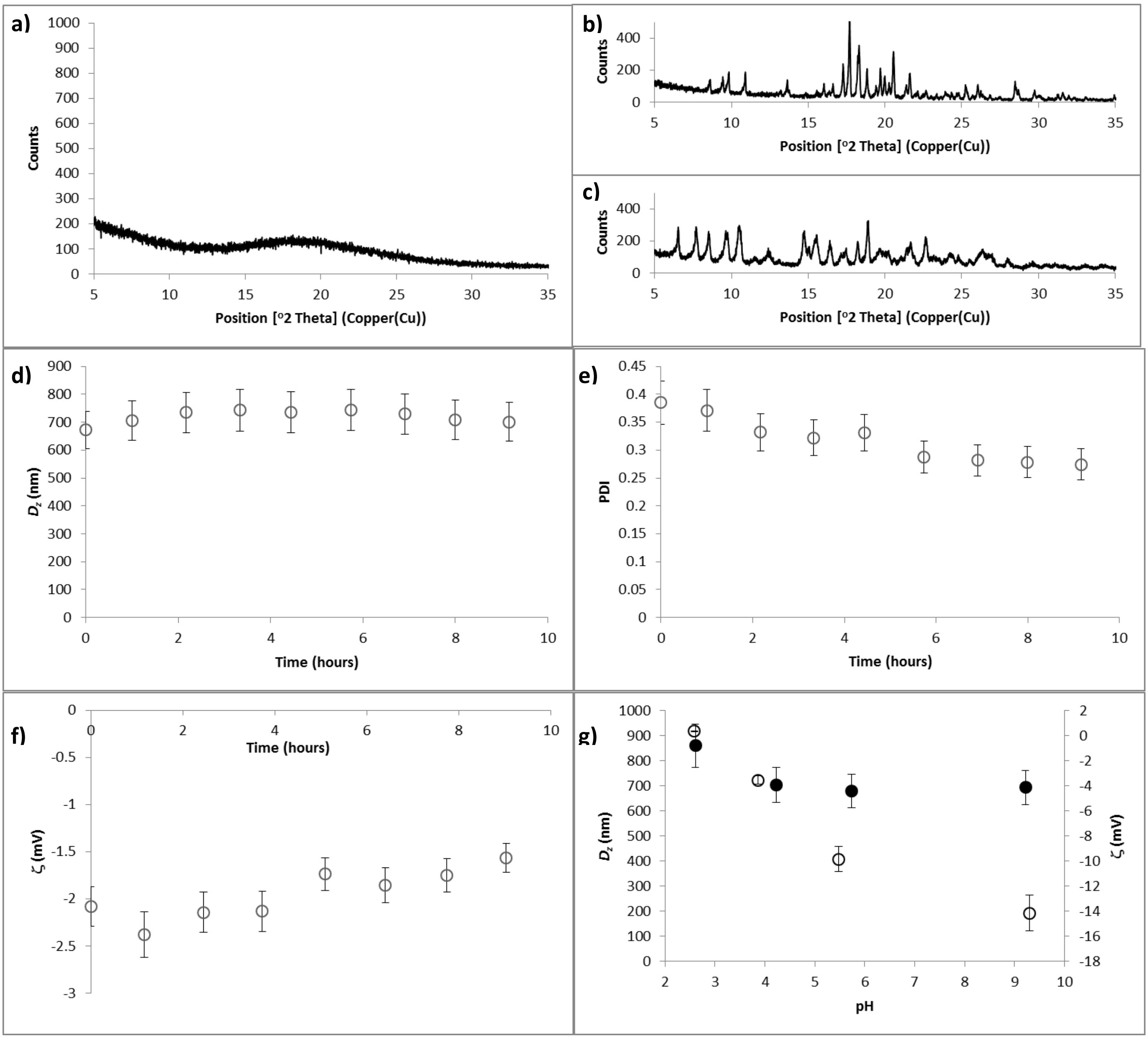

Supplementary Figure 5: Physical characteristics of solid state ETFD monoliths and redispersed samples of the lead LPV/RTV SDN candidate (56 wt% LPV, 14 wt% RTV, 20 wt% PVA, 10 wt% TPGS. a) p-XRD) of the ETFD monolith recorded directly following freeze drying, b) non-processed RTV powder). c) non-processed LPV powder. d) D_z, e) PDI and f) ζ monitoring of ETFD aqueous dispersions; ETFD monoliths were dispersed in water (1 mg mL^{-1}) and monitored via DLS for 10 hours (samples stationary). g) D_z (filled circles) and ζ (open circles) of ETFD dispersions (1 mg mL^{-1} in water) at varied pH. In all cases, samples were studied at 25 ^{o}C and fixed error bars are shown derived from instrumental accuracy. (10% error).

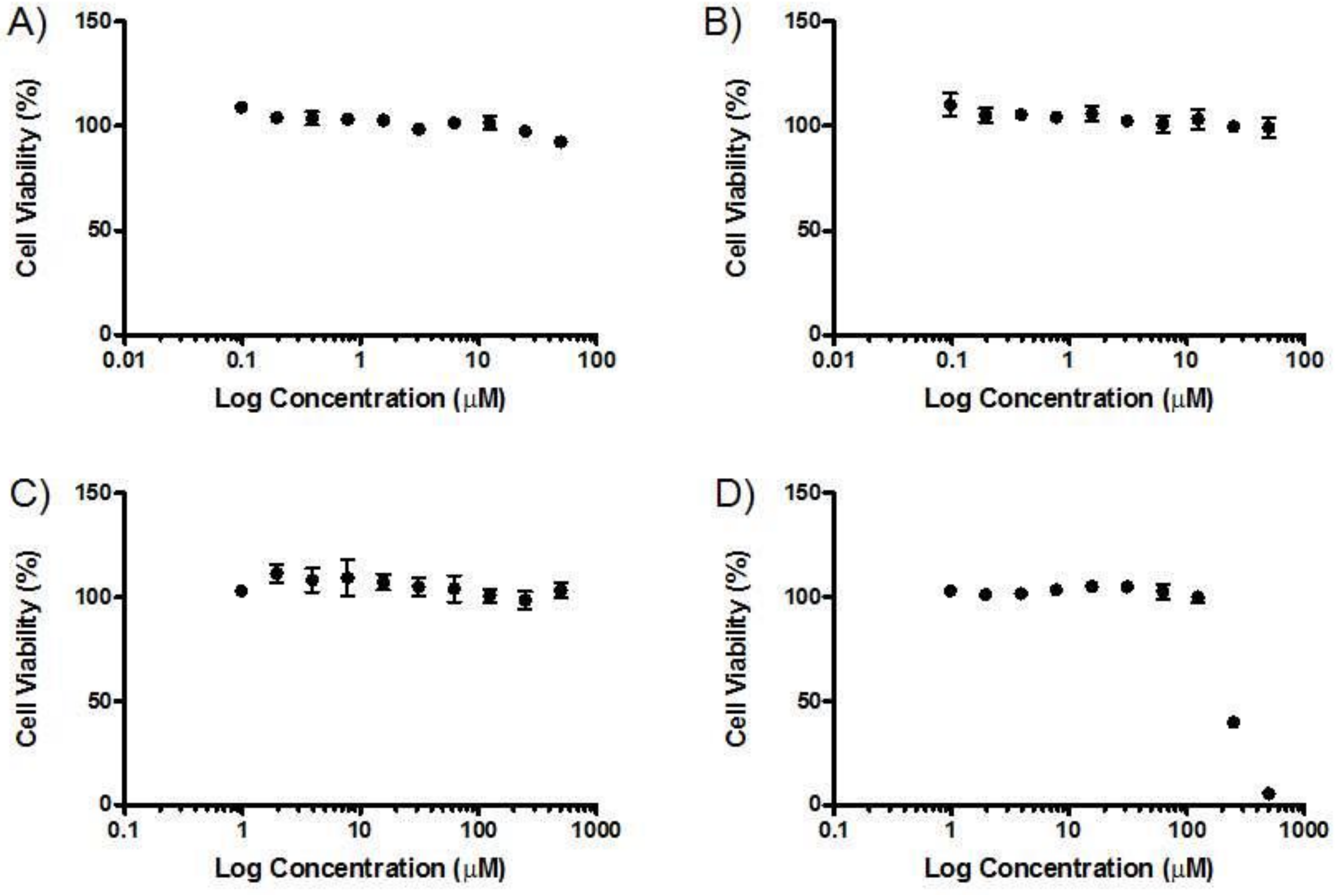

Supplementary Figure 6: Cellular toxicity (Caco-2 cells) of: A) aqueous LPV, B) aqueous dispersion of 50 wt% loaded spray-dried lead LPV SDN, C) aqueous solution of PVA, and D) aqueous solution of TPGS.

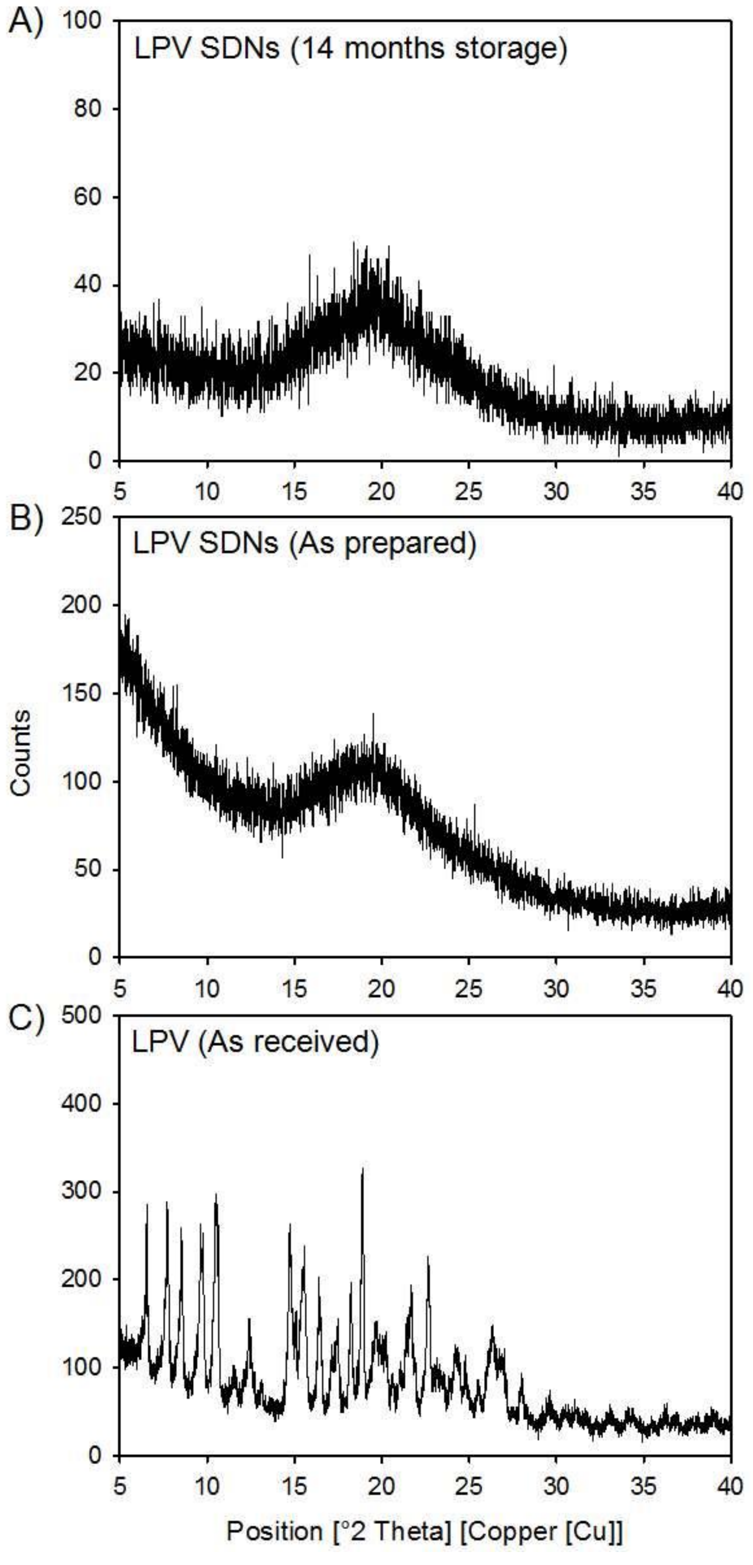

Supplementary Figure 7: Comparative powder X-ray diffraction characterisation of the spray dried lead LPV SDN candidate (50 wt% LPV): A) after 14 months storage under ambient conditions, B) one day after manufacture, and C) LPV as received and prior to SDN manufacture.

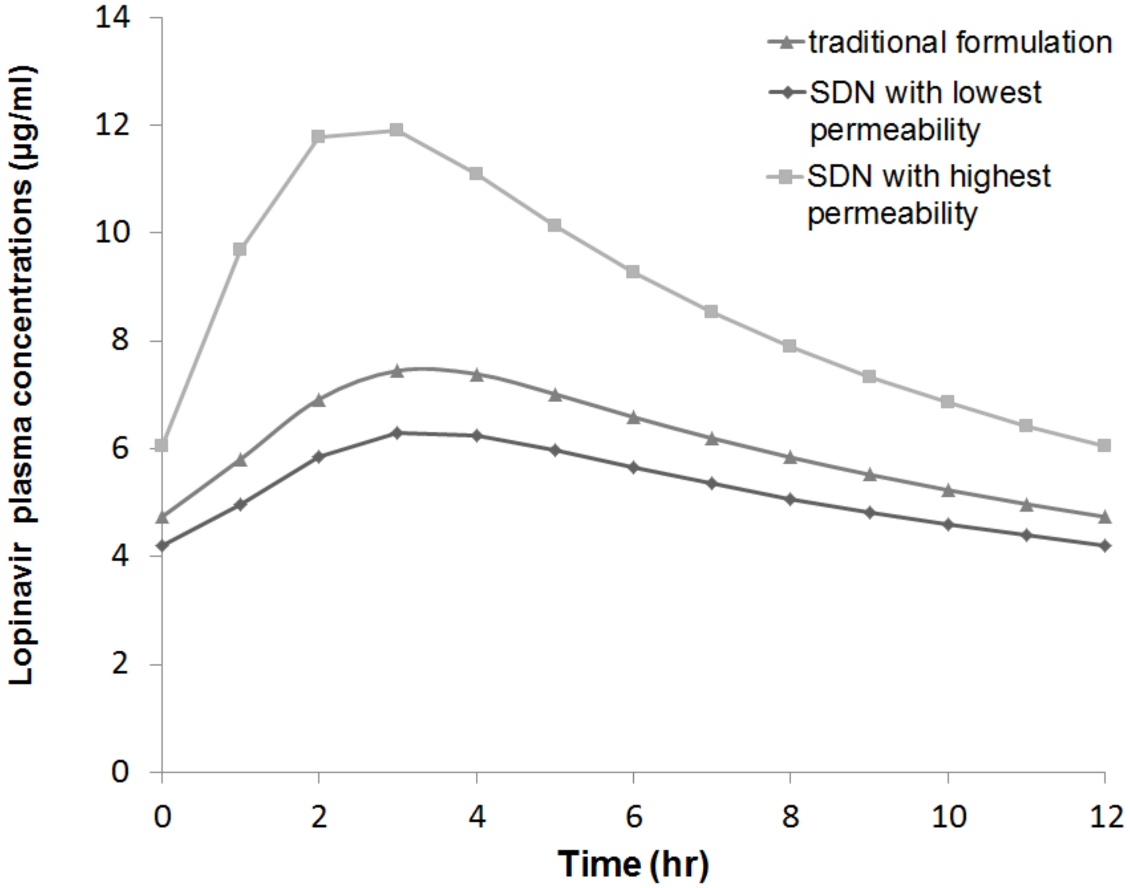

Supplementary Figure 8: Simulation of LPV pharmacokinetics through PBPK modelling. Mean plasma concentrations of LPV in a simulated cohort of 110 virtual individuals receiving 400/100 mg of LPV/RTV twice daily of the LPV aqueous solution (<0.1% DMSO), 10 wt% LPV SDN with the lowest permeability and 10 wt% LPV SDN with the highest permeability.

Supplementary Table 1: Average D_z across 3x 160-component library screens (values in nm, error shows % standard deviation from average D_z across 3 screens) and ζ of second screen (mV). 10% LPV, 60% Polymer, 30% surfactant.

	**PEG 1k**		**F68**		**F127**		**Kollicoat**		**PVA**		**PVP k30**		**HPC**		**HPMC**		**H-Gel**		**NaCMC**	
	D_z	ζ	D_z	ζ	D_z	ζ	D_z	ζ	D_z	ζ	D_z	ζ	D_z	ζ	D_z	ζ	D_z	ζ	D_z	ζ
**Na Alginate**					979±27	-14	1803±19	-19	763±26	-9										
**Na Myristate**															769±15	-28				
**NDC**					525±8	-6	539±9	-5	368±27	-6							676±20	-40		
**Na Caprylate**			421±8	-4	441±9	-2	782±20	-1	407±5	-1			1374±32	-2						
**TPGS**															676±21	-32				
**Sisterna 11**			574±70	-29	605±34	-18	454±16	-20			374±22	-54	419±2	-35	520±8	-24	340±8	-57		
**Sisterna 16**	839±54	-5	489±18	-8			391±22	-2	365±7	-2	293±20	1			868±45	0				
**SDS**					493±15	-24			368±2	-14										
**AOT**									468±9	-10										
**Chremophor EL**	168±9	-7	254±8	-6			1904	-6	220±12	-4	194±22	-1			357±24	-3	180±9	-2	431±3	-6
**Solutol HS 15**	305±30	-5			413±5	-5	234±5	-5	271±28	-2	231±10	0			250±16	2				
**Tween 20**			411±12	-10	422±5	-6	896±106	-3	293±19	-3			614±30	-6	472±20	-4				
**Tween 80**	463±78	-3	369±17	-3	372±15	-4	284±7	-3	260±19	-2	313±20	-2	454±32	-2	309±4	-2	517±57	-4		
**Brij 58**	492±11	-1	537±23	-2	532±36	-1	417±29	-1	300±10	-1					814±31	1	423±21	-1	2018±55	-4
**Hyamine**					636±28	15	733±29	30	412±13	19	579±26	40			668±8	14	350±29	23	1053±49	2
**CTAB**																				

Measured using DLS, 1 mg mL^{-1}, 25°C. Where blank shows sample failed DLS criteria; 1) complete aqueous dispersion; 2) D_z <1000 nm; 3) D_z standard deviation between repeated measurement <10% (n = 3; Fig. 2C&D) and; 4) PDI <0.5.

Supplementary Table 2: Average cellular accumulation ratio (CAR) and cytotoxicity (CC50; μM) in caco-2 cells. 10% LPV, 60% Polymer, 30% surfactant.

	**PEG 1k**		**F68**		**F127**		**Kollicoat**		**PVA**		**PVP k30**		**HPC**		**HPMC**		**H-Gel**		**NaCMC**	
	CAR	CC_{50}	CAR	CC_{50}	CAR	CC_{50}	CAR	CC_{50}	CAR	CC_{50}	CAR	CC_{50}	CAR	CC_{50}	CAR	CC_{50}	CAR	CC_{50}	CAR	CC_{50}
**Na Alginate**					16.4	61.3	23.3	60.2	32.9	167.8										
**Na Myristate**															16.6	62.6				
**NDC**					14.8	104.2	21.7	133.8	15.9	208.5							23.6	165.7		
**Na Caprylate**			13.3		22.1	441.8	27.4	480.1	22.0	312.6			25.3	138.9						
**TPGS**	12.8	73.4	16.1	67.4			22.6	589.3	22.0	288.6	19.5	398.0			22.2	416.8				
**Sisterna 11**					19.9	82.1			30.0	74.7										
**Sisterna 16**									30.8	94.7										
**SDS**															23.9	105.3				
**AOT**			17.2	97.6	18.7	245.8	26.1	208.1			24.9	98.1	22.9	84.6	27.4	134.3	31.0			
**Chremophor EL**	11.0	309.6	17.9	134.4			18.9	321.6	26.3	147.8	24.6	199.3			23.5	176.5	29.8	132.1	31.4	144.8
**Solutol HS 15**	17.5	342.6			17.4	411.3	21.3	146.8	25.1	266.6	22.4	212.1			23.2	289.9				
**Tween 20**			18.2	114.4	17.3	329.6	23.1	94.8	28.8	328.1			24.6	386.4	28.3	211.2				
**Tween 80**	10.0	508.3	16.4	189.4	18.9	402.1	28.0	167.4	27.3	354.0	24.3	188.7	27.2	208.0	29.6	157.1	27.5	123.8		
**Brij 58**	12.2	162.0	17.0	166.0	21.9	201.3	25.1	119.4	25.0	488.8					27.4	344.7	29.0	158.4	36.6	78.6
**Hyamine**					31.4	388.4	26.0	599.7	33.5	376.8	26.3	502.4			35.1	587.7	35.0	188.4	39.6	98.5
**CTAB**																				

Supplementary Table 3: D_z for loaded screens utilising 50% LPV, 40% Polymer, 10% surfactant.

		POLYMERS						
Surfactants		**F68**	**PVP K30**	**HPMC**	**PEG 1k**	**F127**	**Kollicoat**	**PVA**
	**TPGS**			602 ± 5.6			467 ± 2.9	624 ± 2.7
	**Tween 20**							632 ± 1.4
	**Tween 80**			551 ± 3.9			745 ± 4.1	705 ± 1.4
	**Hyamine**			1365 ± 12.4			664 ± 7.7	430 ± 1.0
	**NDC**		1245 ± 4.9	2254 ± 7.9			804 ± 4.7	666 ± 4.1
	**AOT**						687 ± 2.3	719 ± 1.9
	**Solutol HS**			591 ± 17.6			449 ± 14.6	450 ± 2.2

Measured using DLS, 1 mg mL^{-1}, 25°C. Where blank shows sample unable to give DLS data

Supplementary Table 4: D_z for higher loaded screen; 70% LPV, 20% Polymer, 10% surfactant.

		POLYMERS						
Surfactants		**F68**	**PVP K30**	**HPMC**	**PEG 1k**	**F127**	**Kollicoat**	**PVA**
	**TPGS**							700 ±9.5
	**Tween 20**							642 ± 1.6
	**Tween 80**			452 ± 10				590 ± 8.0
	**Hyamine**							
	**NDC**			1127 ± 5.6				659 ± 0.9
	**AOT**							
	**Solutol HS**			377 ± 0.7				

Measured using DLS, 1 mg mL^{-1}, 25°C, Where blank shows sample unable to give DLS data

Supplementary Table 5: D_z for variation of surfactant/polymer ratio; 70% LPV constant.

	**5% Surfactant 25 % Polymer**	**10% Surfactant 20 % Polymer**	**15% Surfactant 15 % Polymer**	**20% Surfactant 10 % Polymer**
**PVA / TPGS**	608 ± 4.9	516 ± 0.3	605 ± 1.9	688 ± 3.9
**PVA / T-20**	621 ± 2.4	656 ± 1.9	660 ± 2.1	778 ± 3.0
**PVA / T-80**		523 ± 3.2	637 ± 1.1	902 ± 0.3
**HPMC / T-80**	746 ± 1.4	518 ± 3.0	614 ± 10.4	739 ± 1.8
**PVA / NDC**	617 ± 5.9	595 ± 1.9	653 ± 2.9	
**HPMC / NDC**	839 ± 6.4	891 ± 7.1	970 ± 6.1	1390 ± 6.3
**HPMC / Solutol**		485 ± 4.2		

Measured using DLS, 1 mg mL^{-1}, 25°C. Where blank shows sample unable to give DLS data

Supplementary Table 6: Optimised formulations for LPV solid drug nanoparticles (LPV 70 wt%).

SDN #	Polymer (wt%)	Surfactant (wt%)	D_z^a [nm]	PDI[a]	ζ[a] [mV]
1	PVA (25)	TGPS (5)	589 ± 28	0.40 ± 0.13	-11 ± 7
2	PVA (25)	Tween 20 (5)	600 ± 30	0.36 ± 0.03	-15 ± 9
3	PVA (25)	NaDC (5)	645 ± 39	0.33 ± 0.03	-11 ± 4
4	PVA (20)	TGPS (10)	566 ± 26	0.37 ± 0.02	-12 ± 2
5	PVA (20)	Tween 20 (10)	984 ± 23	0.53 ± 0.14	-18 ± 0.7
6	PVA (20)	NaDC (10)	701 ± 47	0.43 ± 0.05	-12 ± 0.1

Measured using DLS, 1 mg mL^{-1}, 25°C

Supplementary Table 7: Optimised formulations for LPV/RTV solid drug nanoparticles (LPV/RTV 56/14 wt%).

SDN#	Polymer (wt%)	Surfactant (wt%)	LPV/RTV Ratio	D_z [nm]	PDI	ζ [mV]
1	PVA (25)	TGPS (5)	4/1	906 ± 39	0.34 ± 0.06	0 ± 0.5
			8/1	585 ± 9	0.33 ± 0.03	
			40/1			
2	PVA (25)	Tween 20 (5)	4/1	684 ± 77	0.32 ± 0.05	-3 ± 1.2
			8/1	599 ± 13	0.42 ± 0.02	
			40/1			
3	PVA (25)	NaDC (5)	4/1	661 ± 34	0.22 ± 0.01	-12 ± 0.5
			8/1			
			40/1			
4	PVA (20)	TGPS (10)	4/1	615 ± 91	0.32 ± 0.06	-4 ± 1.6
			8/1	654 ± 12	0.36 ± 0.03	
			40/1	556 ± 17.41	0.46 ± 0.1	-10.5 ± 0.5
5	PVA (20)	Tween 20 (10)	4/1	715 ± 20	0.39 ± 0.01	-8 ± 0.9
			8/1	891 ± 33	0.41 ± 0.05	
			40/1			
6	PVA (20)	NaDC (10)	4/1	662 ± 54	0.26 ± 0.02	-14 ± 0.5
			8/1			
			40/1			

Measured using DLS 1 mg mL^{-1}, 25 ^{o}C. Dash shows data not measured.

Supplementary Table 8: Transcellular permeation of 70 wt% loaded LPV SDNs across Caco-2 cells.

SDN #	Time (hours)	Apical to Basolateral permeation (mean ± SD; nmol)	Basolateral to Apical permeation (mean ± SD; nmol)
**Aqueous control**	1	50.6 ± 6.3	249.6 ± 27.0
	2	253.8 ± 37.7	384.4 ± 96.7
	3	359.4 ± 38.7	566.1 ± 129.7
	4	522.6 ± 19.2	636.5 ± 86.0
**1**	1	165.2 ± 13.3	216.6 ± 74.4
	2	134.8 ± 26.2	346.6 ± 18.7
	3	212.3 ± 76.9	484.6 ± 54.0
	4	479.2 ± 103.5	968.1 ± 71.2
**2**	1	138.1 ± 40.2	196.8 ± 97.0
	2	192.9 ± 8.5	491.2 ± 20.5
	3	352.8 ± 81.0	1053.9 ± 98.4
	4	241.0 ± 57.5	661.9 ± 57.7
**3**	1	125.1 ± 29.0	338.8 ± 102.5
	2	180.0 ± 33.5	487.5 ± 18.1
	3	292.8 ± 83.0	829.1 ± 98.8
	4	316.6 ± 47.2	846.3 ± 130.5
**4**	1	239.9 ± 30.6	418.7 ± 85.6
	2	418.4 ± 72.8	504.4 ± 60.5
	3	553.4 ± 64.5	997.2 ± 119.5
	4	606.6 ± 72.3	1172.5 ± 67.0
**5**	1	166.7 ± 26.9	213.8 ± 70.3
	2	290.9 ± 21.2	453.8 ± 70.7
	3	448.4 ± 43.7	779.4 ± 43.4
	4	510.5 ± 44.7	855.3 ± 55.3
**6**	1	150.6 ± 13.0	120.6 ± 36.5
	2	235.1 ± 28.1	728.1 ± 86.2
	3	301.6 ± 85.5	854.0 ± 101.8
	4	373.5 ± 47.6	936.9 ± 143.2

Supplementary Table 9: Transcellular permeation of 50 wt% loaded LPV/RTV combination SDNs across Caco-2 cells.

Formulation	Time (hours)	Apical to Basolateral permeation (mean ± SD; nmol)	Basolateral to Apical permeation (mean ± SD; nmol)
**Aqueous control LPV/RTV 1:4**	1	77.6 ± 4.5	70.8 ± 17.9
	2	136.2 ± 63.7	99.5 ± 10.2
	3	178.9 ± 49.5	125.0 ± 4.6
	4	176.3 ± 58.3	151.5 ± 7.3
**Aqueous control LPV/RTV 1:10**	1	56.9 ± 7.9	60.2 ± 6.8
	2	92.1 ± 11.5	92.3 ± 4.4
	3	120.2 ± 27.5	124.5 ± 13.6
	4	124.5 ± 29.3	118.6 ± 6.4
**Aqueous control LPV/RTV 1:40**	1	44.1 ± 13.7	48.0 ± 6.8
	2	63.7 ± 21.7	78.0 ± 10.0
	3	74.4 ± 32.6	112.1 ± 19.2
	4	79.2 ± 36.1	95.8 ± 11.6
**SDN LPV/RTV 1:4**	1	380.9 ± 33.3	374.5 ± 69.4
	2	684.0 ± 91.5	689.6 ± 38.3
	3	895.9 ± 401.2	931.8 ± 77.7
	4	922.5 ± 348.7	1104.5 ± 78.6
**SDN LPV/RTV 1:10**	1	545.3 ± 507.0	763.7 ± 849.9
	2	704.6 ± 561.5	669.4 ± 51.5
	3	815.8 ± 510.5	1009.7 ± 118.9
	4	872.5 ± 653.7	1112.1 ± 212.0
**SDN LPV/RTV 1:40**	1	356.7 ± 22.5	373.3 ± 68.1
	2	578.2 ± 38.9	617.4 ± 62.5
	3	767.5 ± 36.3	821.8 ± 118.0
	4	854.9 ± 53.9	1040.9 ± 86.3

Supplementary Table 10: Permeability data for 50 wt% loaded spray-dried lead LPV through a triple culture model.

**Cell layer condition**	**Time (hours)**	**Apical to Basolateral permeation (mean ± SD; nmol)**
**Caco-2**	1	190.7 ± 24.9
	2	394.9 ± 50.3
	3	616.2 ± 58.7
	4	758.0 ± 54.9
**Caco-2 plus M cells**	1	155.5 ± 36.0
	2	345.9 ± 56.7
	3	538.3 ± 61.3
	4	679.5 ± 69.6
**Caco-2 plus HT29 cells**	1	90.3 ± 8.5
	2	204.4 ± 31.8
	3	352.9 ± 52.0
	4	481.2 ± 59.8
**Caco-2 plus M cells and HT29 cells**	1	101.9 ± 21.1
	2	249.0± 51.2
	3	413.8 ± 65.0
	4	540.1 ± 93.3

Supplementary Table 11: LPV drug product stability specification.

	Assay<br>(LPV mg/capsule)	Total Related Substances<br>(% Area normalization)<br>Authorised USP Pending Monograph Version 1; Lopinavir. v.1 authorised September 1, 2009
Specification	90 – 110% of label claim<br>(Label claim 150 mg LPV)	Total impurities NMT 1.0%<br>Any individual unknown impurity NMT 0.1%

Supplementary Table 12: Drug product stability data (150 mg LPV capsules).

Time Point	Storage Conditions	Assay<br>(mean mg $capsule^{-1}$)	Total Related Substances<br>(% Area normalization)	Comments
Initial	N/A	154.10 mg	0.19%	In line with specification
1 Month	25°C/60%RH	149.95 mg	0.22%	In line with specification
	40°C/75%RH	156.94 mg	0.21%	
3 Month	25°C/60%RH	151.90 mg	0.32%	In line with specification
	40°C/75%RH	145.29 mg	0.37%	
6 Month	25°C/60%RH	157.12 mg	0.15%	In line with specification
	40°C/75%RH	155.05 mg	0.14%	
12 Month	25°C/60%RH	137.70 mg	0.11%	In line with specification

Specification refers to those stated in Table S.11, taken from Authorised United States Pharmacopeia (USP) Pending Monograph Version 1; Lopinavir. v.1 authorised September 1, 2009

Supplementary Methods

Materials were purchased or donated from various suppliers/companies as follows: BASF (Royal Tunbridge Wells, UK) - polyethylene glycol15 –hydroxystearate (Solutol® HS 15), macrogol glycerol ricinoleate (Cremophor® EL), PVA– graft -poly(ethylene glycol) copolymer (Kollicoat®) and Kolliphor TPGS; Fluka Chemicals (Dorset, UK) - sodium 1,4-bis(2-ethylhexoxy)-1,4- dioxobutane-2-sulfonate (AOT), octanoic acid sodium salt (Na Caprylate) and poly(vinylpyrrolidone) (PVP K30); Sisterna® (Roosendaal, The Netherlands) - sucrose stearate SP70 (Sisterna® 16), sucrose stearate SP50 (Sisterna® 11); Merck KGaA (Darmstadt, Germany) - polyvinyl alcohol (PVA grade 4-88, MW 57-77,000); Fisher Scientific (Loughborough, UK) - chloroform, dichloromethane and ethanol; LGM Pharma (Chicago, US) and donated by CIPLA (Mumbai, India) - lopinavir and ritonavir; Moravek Biochemicals, Inc (Brea, US) 3H-radiolabeled lopinavir; American Type Culture Collection (ATCC; USA) - caco-2 cells; Nunclon (Denmark) - 96-well plates; Promega (UK) - CellTiter-Glo cell viability assay; Perkin Elmer (UK) - Ultima Gold scintillation fluid; Meridian Biotechnologies Ltd (UK) -scintillation vials; Wallac (UK) – Optisolve. All other reagents were purchased from Sigma-Aldrich (Dorset, UK).

Dynamic light scattering (DLS) characterization of aqueous LPV Nanodispersions: Immediately prior to analysis, samples were dispersed by addition of water (1 mg mL^{-1} with respect to API) and vortex mixed to generate a uniform dispersion. Z-average diameter (D_z), zeta potential (ζ), polydispersity index (PDI) and number average diameter (D_n) were determined at 25 °C using a Malvern Zetasizer Nano ZS equipped with a 4 mW He–Ne, 633 nm laser, and using plastic disposable cuvettes. Malvern Zetasizer software version 7.03 was used for data analysis. ζ measurements carried out at an initial pH of 6.5, using disposable capillary zeta cells. All data is reported as an average of three measurements using automatic optimization settings.

Powder X-ray diffraction (XRD) measurements were collected in transmission mode on solid monolith or powder samples held on a thin Mylar film in aluminium well plates on a Panalytical X'Pert PRO MPD instrument with X'Pert Operator Interface (version 1.0b) software. The instrument utilizes a high throughput screening XYZ stage, X-ray focusing mirror, and PIXcel detector, using Ni-filtered Cu K α radiation. Data were measured over the range 4–50° in ≈ 0.013° steps over 60 min.
